# Indicators of nutritional risk in hospital inpatients: a narrative review

**DOI:** 10.1017/jns.2021.86

**Published:** 2021-12-10

**Authors:** Alison Yaxley, Reegan K. Knowles, Sebastian H. Doeltgen, Diane J. Chamberlain, Raechel A. Damarell, Michelle D. Miller

**Affiliations:** College of Nursing and Health Sciences, Flinders University, PO Box 2100, Adelaide, SA 5001, Australia

**Keywords:** Acute care, Adult, Indicators, Inpatients, Nutritional risk, Phrases, Review

## Abstract

Malnutrition is common in the acute care setting. Despite the existence of a plethora of screening tools, many malnourished patients remain undiagnosed and untreated, in part due to competing responsibilities for screening staff, under- or over-referral to dietetics services, and inadequate dietetics resources. Better identification of patients at risk of malnutrition would enable optimised care provision and streamlined care pathways. This narrative review of reviews aimed to collate and synthesise literature documenting nutritional risk factors in adult hospital inpatients, to generate a comprehensive list of nutritional risk indicators from high methodological quality review articles. Six electronic databases were searched (Medline, Cumulative Index to Nursing and Allied Health Literature, Cochrane Database of Systematic Reviews, Joanna Briggs Institute Database, Embase and Scopus) using a systematic search strategy. Three researchers screened the resulting 5889 citations, identifying 59 reviews summarising original studies that investigated associations between indicators and measures of malnutrition, undernutrition or nutritional risk. After quality appraisal by two researchers, using the American Dietetic Association Quality Criteria Checklist for Review Articles, seven reviews were classified as high quality, identifying fifty-seven unique indicators of nutritional risk (disease status/condition – twenty-three; eating/appetite/digestion – twelve; type of diet – five; cognition/psychology/social factors – five; medication-related – two; miscellaneous – ten). This is the first comprehensive list of nutritional risk factors in adult hospital inpatients, derived from only the highest methodological quality reviews. This list contributes to the development of practice and evidence-informed systems-level approaches to the identification of nutritional risk in the acute care setting.

## Graphical abstract

**Figure:**
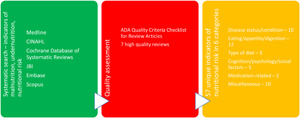

## Introduction

Malnutrition is common in the acute care setting with an estimated prevalence of 20–50%, depending on the method of diagnosis and specific patient group^(1)^. Malnutrition can exist pre-admission, worsen during admission and/or develop during a hospital stay^(1)^. The presence of malnutrition is an independent risk factor for a range of negative patient outcomes, including increased length of hospital stay^(2)^, poor wound healing, functional decline and adverse psychosocial outcomes^(3)^. In addition to poor patient outcomes, it has been estimated that malnutrition costs the global economy US$3⋅5 trillion per year^(4)^.

In order to implement timely and appropriate nutritional support, dietitians can conduct a comprehensive clinical assessment, or complete one of several validated malnutrition assessment tools, e.g. the Subjective Global Assessment^(5)^, to thoroughly investigate a patient's nutritional status and determine their individual nutritional needs. However, the time-consuming and resource-intensive nature of nutritional assessment often means that not all patients can be assessed during their hospital stay. Therefore, a number of malnutrition screening tools have been developed to quickly identify patients at risk of malnutrition and enable timely referral for comprehensive assessment by dietetics staff^(6)^. These tools may be performed by any healthcare worker, relieving workforce burden on dietitians. Commonly, they focus on only a few markers of nutritional status, such as BMI, weight loss and clinical condition, e.g. the Malnutrition Universal Screening Tool (MUST)^(7)^.

Despite the high prevalence of malnutrition, its serious consequences and the number of screening tools available to identify at-risk patients, malnutrition in the acute setting often remains underdiagnosed and undertreated^(7)^. Proposed reasons for this include inconsistent policies related to malnutrition screening^(8)^, competing priorities of hospital staff and lack of adequate staff training^(9,10)^. Furthermore, even patients that are screened for malnutrition may not be identified as being at risk because the need for screening tools to be simple and time effective does not allow for consideration of a broad range of risk factors, or for objective measurement of factors influencing nutritional risk. Finally, even if patients are flagged as at risk, they may not be assessed by a dietitian despite positive screening and referral, as limited dietetic resources necessitate prioritisation of cases^(11)^. This issue is compounded by over-referral due to the lack of sensitivity of some screening tools^(12)^. The significant limitations in the existing malnutrition screening, assessment and diagnosis processes result in suboptimal identification and management of malnutrition in acute care settings.

One of the main limitations of contemporary malnutrition screening tools is that they do not consider the large range of nutritional risk factors outside of the physical and anthropometric measures commonly considered by existing tools. Such factors include aspects of the hospital environment, surgery status, behavioural difficulties, support at meal time, therapeutic diets (e.g. nil-by-mouth, pureed, etc.), and food options and taste preferences^(13)^. In addition, multiple indicators may combine to influence relative risk on an individual basis. However, to date, no systematic interrogation of the literature has been undertaken to identify and collate previously documented indicators of nutritional risk. A comprehensive understanding of all the factors identified to date which modulate malnutrition risk would provide a basis from which novel, innovative, systems-based screening pathways could be developed that more comprehensively and holistically assess an individual patient's risk for malnutrition. In an era of individualised, patient-centred and budget-conscious health care, such novel triage systems would contribute to optimised referral, assessment and care pathways.

In order to collate and synthesise existing literature documenting nutritional risk factors, we performed a narrative review of reviews, utilising a systematic literature search strategy, to investigate the association between indicators and nutritional risk in adult inpatients.

## Methods

### Registration

This narrative review has been registered (PROSPERO 2018 CRD42018105464).

### Data sources and search strategy

An experienced research librarian (RD) searched Medline (Ovid), Cumulative Index to Nursing and Allied Health Literature (CINAHL), Cochrane Database of Systematic Reviews, Joanna Briggs Institute Database, Embase (Ovid) and Scopus for reviews in the English Language, published up to May 2019. A large number of search terms for each concept of relevance to the topic were used to locate studies. These concepts were the acute setting (hospitalised, inpatient), risk in general (risk, determinants, predisposing factors) and nutritional risk status specifically (malnutrition, vitamin deficiency). The search strategy was first developed for Ovid Medline (Supplementary Table S1) and then translated for each of the other target databases. Reference lists of selected articles were hand-searched to identify further articles.

### Screening and review selection

After removal of duplicates, the articles located were imported into Covidence, an online software program that facilitates study selection^(14)^. At least two researchers (AY, MM or RK) independently examined titles and abstracts to select articles for full-text screening. Conflicts were resolved by discussion between the original screeners or by a third researcher. Relevant review articles were obtained in full text. Two researchers (AY and RK) independently examined the full-text articles against the inclusion and exclusion criteria. Again, inter-reviewer disagreement on inclusion of particular articles was resolved through discussion.

#### Inclusion criteria

- English language;
- Type of study: Review articles that summarised any type of study design, including RCTs, observational studies, case-control or other quasi-experimental studies;
- Study populations: Adult (aged ≥18 years) hospital inpatients; and
- Review articles summarising original studies that investigated an association between an indicator and a measure of malnutrition, undernutrition or nutritional risk.

#### Exclusion criteria

All other articles as well as reviews not meeting pre-defined criteria for methodological quality.

### Quality appraisal

Two reviewers (AY and RK) appraised the quality of included articles as being either positive, negative or neutral, using the American Dietetic Association (ADA) Quality Criteria Checklist for Review Articles^(15)^. Given the large number of reviews (and therefore indicators) located, and the objective to focus on indicators supported by high-quality evidence, only reviews classified as positive (indicating that the report clearly addressed issues of inclusion/exclusion bias, generalisability, and data collection and analysis) were included in this qualitative synthesis.

### Data extraction

For each included review, one researcher (RK) extracted from the full text those risk indicators that were assessed as being associated with at least one of the outcome variables (i.e. malnutrition, undernutrition or nutritional risk).

## Results

**Fig. 1.**
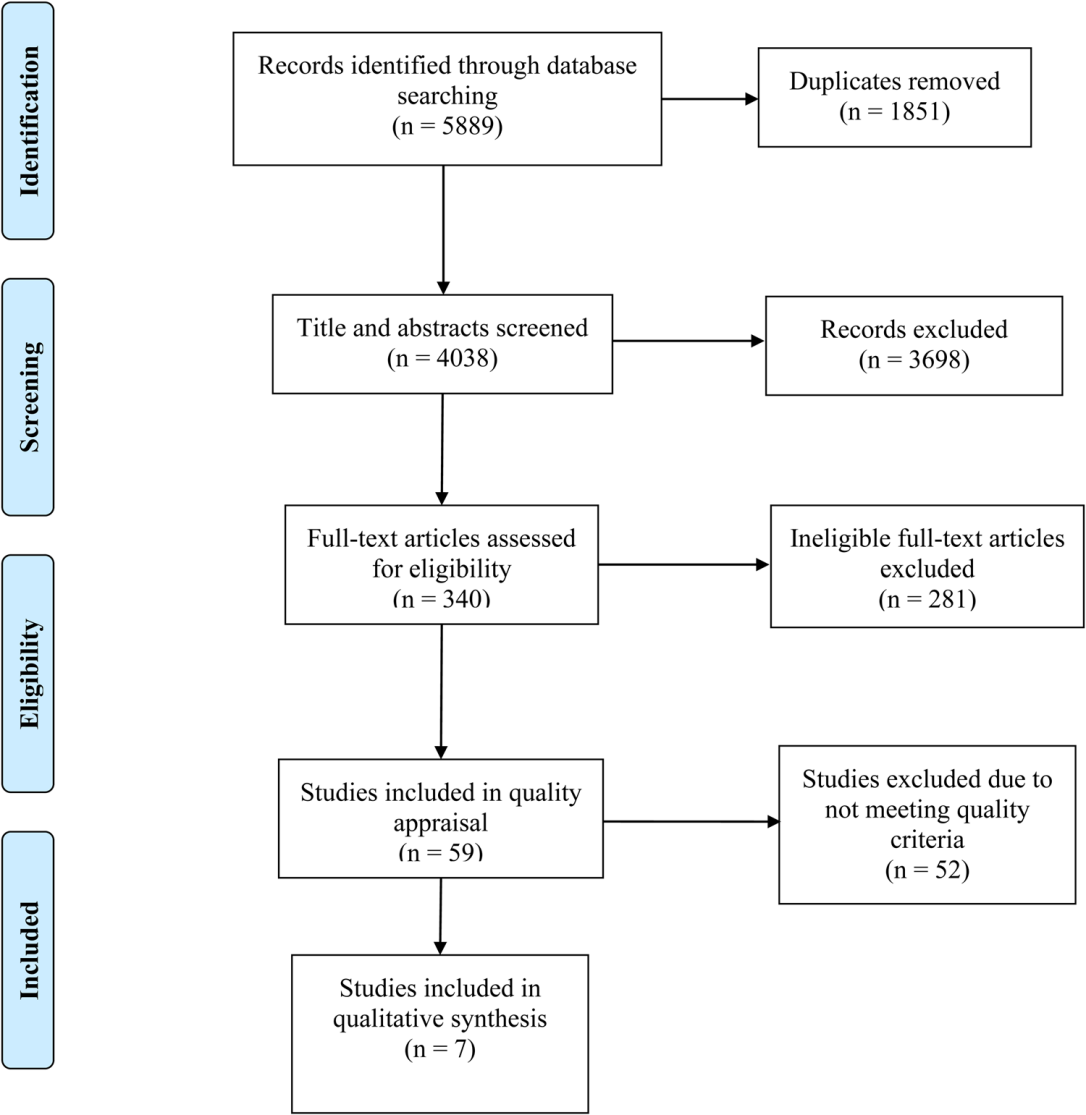
Flowchart of the findings of the search and screening process for a literature review to identify reviews reporting nutritional risk indicators in adult acute care inpatients.

The initial literature search yielded a total of 5889 citations (see Fig. 1). Following the removal of duplicates, title, abstract and full-text screening, fifty-nine reviews were selected for quality appraisal. Seven articles were identified as ‘positive’ according to the ADA Quality Criteria Checklist for Review Articles and therefore included in the current review^(13,16–21)^.

Fig. 1 shows that fifty-two potentially eligible reviews were excluded due to not meeting quality criteria. The most common limitations identified across the excluded reviews were that the processes for extraction, synthesis and analysis of data were not clearly described, and quality appraisal of articles was not completed or was not clearly reported. In addition, biases and limitations did not appear to have been taken into consideration in the conclusions reported. The seven high-quality studies included in the final analysis were assessed using the NHMRC hierarchy of evidence^(22)^, and considered Grade 1. This is the highest grade of research evidence and commonly used to suggest that evidence can be trusted to guide practice.

From the seven included reviews, a total of fifty-seven unique indicators were identified as being associated with nutritional risk (see Table 1). Twenty-three of these indicators are related to a disease status or condition (stroke (seven separate indicators were identified regarding stroke, i.e. stroke with several associated conditions/circumstances were investigated), cancer, psoriasis, acute pancreatitis, Parkinson's disease, chronic alcoholism, depression, dementia, ear, nose and throat disease, infectious disease, inflammatory disease, organ failure, gastrointestinal disease, neurodegenerative disease, vascular disease, fracture and oropharyngeal candidiasis); twelve are related to eating, appetite or digestion (dysphagia, dysgeusia, anorexia, dental hygiene, dentures, chewing, dry mouth, reduced food intake, eating impairment, malabsorption, maldigestion and severe constipation); five were types of diets (slimming, diabetic, residue-free, salt-free and cholesterol-lowering); five describe altered cognition/psychology or social factors (drowsiness, delirium, altered level of consciousness, grieving and social isolation); two are medication-related (polypharmacy, long-term use of corticosteroids) and remaining indicators include energy requirements, pressure sores, pain, financial difficulties, ill-treatment, lifestyle change, behavioural disorders, adductor pollicis muscle thickness, surgery and mobility impairment. Stroke-related dysphagia was the only indicator identified in more than one review, and dysphagia independent of stroke was also identified by one study.

**Table 1.** Characteristics of articles included in a review of reviews reporting nutritional risk indicators in adult acute care inpatients

Reference, country	Search range	Population (age)	Number of studies/participants	Study designs of included studies	Nutrition-related outcome variable (measure)	Nutritional risk indicators/positively associated with malnutrition (effect size where reported)
Chen *et al.*^(16)^, China	January 1990 to September 2017	Adult stroke patients (age not reported)	29/8838	Cohort (*n* 19); Cross-sectional (*n* 8); Case-control (*n* 1)	Malnutrition (papers include if used any definite parameter to assess nutritional status)	Stroke patient with malnutrition on admission (OR 8⋅34, 95 % CI: 4⋅60, 15⋅10, *P* < 0⋅00001) Stroke patient with dysphagia (OR 2⋅60, 95 % CI: 2⋅24, 3⋅03, *P* < 0⋅00001) Stroke patient with previous stroke history (OR 3⋅04, 95 % CI: 2⋅35, 3⋅95, *P* < 0⋅00001) Stroke patient with diabetes mellitus (OR 1⋅79, 95 % CI: 1⋅35, 2⋅38, *P* < 0⋅0001) Stroke patient being tube fed (OR 5⋅43, 95 % CI: 3⋅99, 7⋅37, *P* < 0⋅00001) Stroke patient experiencing reduced level of consciousness (OR 2⋅82, 95 % CI: 2⋅12, 3⋅75, *P* < 0⋅00001)
Fruchtenicht *et al.*^(17)^, Brazil	December 1994 to December 2014	Critically ill adult inpatients with cancer (age not reported)	6/1151	Cross-sectional (*n* 4); Case-control (*n* 1); Cohort (*n* 1)	Nutrition status (biochemical measures, weight, BMI, indirect calorimetry, PG-SGA)	Cancer
Lew *et al.*^(18)^, Singapore	To May 2015	Hospital inpatients (>18 years)	9/1777	Cross-sectional (*n* 7); Cohort (*n* 2)	Malnutrition (Subjective Global Assessment)	Adductor pollicis muscle (area under ROC curve: 0⋅92 = excellent discrimination; high specificity: 97⋅8–100 %; kappa: 0⋅04–0⋅25 = poor concordance)
Miller *et al.*^(19^ Denmark	To October 2012	Adults with psoriasis and controls (age not reported)	75/30 190 380	Not reported	Dyslipidaemia	Psoriasis (OR 1⋅5; 95 % CI: 1⋅4, 1⋅7)
Perry *et al.*^(20)^, UK	To September 1999	Adults experiencing acute stroke (age not reported)	Twenty-six studies included in review, three studies investigated association between stroke-related conditions (i.e. dysphagia and aspiration) and nutrition status/293 (from three studies)	Cross-sectional (*n* 2) Longitudinal (*n* 1)	Nutrition status (time to return to pre-stroke diet, anthropometric measures, biochemical measures, calorie-nitrogen deficit)	Stroke-related dysphagia Stroke-related aspiration
Raynaud-Simon *et al.*^(13)^, France	Not reported	Elderly (>70 years)	Not reported	Not reported	Malnutrition	Chronic Alcoholism Malabsorption Dysphagia Polypharmacy Maldigestion Parkinson's Disease Dysgeusia Dementia Infectious Disease Organ Failure (cardiac, respiratory, renal or hepatic) Inflammatory Disease Gastrointestinal Disease Energy Requirement Depression Neurodegenerative Disease Vascular Disease Pressure Sores Anorexia Drowsiness Delirium Behavioural Disorders Dry Mouth Reduced Food Intake Social Isolation Chewing Dental Hygiene Denture Ear, Nose, Throat Disease Grieving Financial Difficulties Ill-Treatment Lifestyle Change Altered Level of Consciousness Long-term Corticosteroid Therapy Pain Surgery Eating Impairment Mobility Impairment Salt-free Diet Slimming Diet Diabetic Diet Cholesterol-lowering Diet Residue-free Diet Severe Constipation Fracture causing a Disability Oropharyngeal Candidiasis
Tenner *et al.*^(21)^, USA	1966–2012	Adult inpatients with acute pancreatitis (age not reported)	Not reported	Not reported	Malnutrition	Acute pancreatitis

*Abbreviations*: *n*, number; OR, Odds Ratio; CI, Confidence Interval; P, *P*-value; BMI, Body Max Index; PG-SGA, Patient-Generated Subjective Global Assessment; ROC, Receiver Operator Characteristic.

Included articles were published between 2001 and 2017 in seven different countries (France^(13)^, China^(16)^, Brazil^(17)^, Singapore^(18)^, Denmark^(19)^, the UK^(20)^ and the US^(21)^). Two reviews did not report the number of original articles reviewed or the total number of participants^(13,21)^, however across the remaining reviews, a total of 122 original articles were included, with a total of 30 202 439 participants. Although not all reviews reported the study designs of the included original research, those that did indicated that they were all observational designs, including cohort, case-control and cross-sectional designs. All reviews investigated the association between potential indicators and malnutrition in adult hospital inpatients, with five focusing on the relationship in cohorts with a diagnosed disease (two cohorts had experienced stroke^(16,20)^, one cancer^(17)^, one acute pancreatitis^(21)^ and one psoriasis^(19)^). Reynaud-Simon and colleagues^(13)^ investigated indicators in the elderly hospitalised (>70 years) population, while Lew and colleagues^(18)^ included all hospitalised adults. Across the reviews, a range of outcomes were measured as proxy indicators of malnutrition/nutritional status, with some reviews not specifying these outcome measures^(13,16,21)^. The measures in the remaining four papers included biochemical measures^(17,19,20)^, weight, BMI and anthropometric measures^(17,20)^, time to return to normal diet^(20)^, malnutrition assessment tool^(17,18)^ and indirect calorimetry^(17)^.

## Discussion

While there is a plethora of literature reporting on nutritional risk and malnutrition, there is a need to be confident in the quality of the published evidence to guide clinical decision making. This narrative review of reviews is the first to extract a comprehensive list of nutritional risk factors in adult acute care inpatients, from only the highest quality review articles, which will contribute to the development of automated practice and evidence-informed systems-level approaches to the identification of nutritional risk in this setting.

Following a systematic literature search strategy, seven reviews of high methodological quality presenting evidence for fifty-seven indicators of nutritional risk were identified. This narrative review has highlighted the lack of high-quality evidence in this area. Nonetheless, the large number of indicators identified demonstrates that maintenance of adequate nutrition is highly multifactorial and influenced by a wide range of diseases and symptoms, personal circumstances, environments, treatments and psychological factors. Interestingly, there was little overlap of risk factors between the included reviews, with stroke being the only factor reported to be associated with malnutrition in more than one study. It is possible that further overlap was present in reviews not included in this narrative review, but our aim was to identify only those indicators presented in high-quality reviews. Furthermore, given that only reviews were included in this narrative review, this suggests that there are likely multiple original studies with consistent findings.

Many of the risk factors identified in this review of reviews are not included in existing, commonly used screening tools and extend beyond the biochemical and anthropometrical measures typically assessed, e.g. the MUST includes body mass index, weight loss and acute disease effect^(7)^, while the Malnutrition Screening Tool includes only unintentional weight loss and poor intake related to decreased appetite^(23)^. It is possible that these tools were developed before the high-quality evidence considered in this review was available, or that practical limitations guided the selection of the assessment items included in these screening tools. It is evident from the findings of our review of high-quality reviews, however, that existing tools may not truly identify those at risk of malnutrition. We propose that our findings provide a timely opportunity to revise and re-orient current malnutrition identification and management processes, by automated means.

We acknowledge the need for screening tools to be able to be rapidly conducted and that the large number of risk factors identified in the current review is unlikely translatable into another manual screening tool of this type. We foresee that it is more likely that the vast amount of information required to screen for all of the identified risk factors will need to be processed by automated, systems-based algorithms. The development of an automated system that capitalises on the advantages of artificially intelligent machine learning could overcome the otherwise prohibitively time-consuming screening process. A recent scoping review of technology used to identify hospital malnutrition identified few screening tools and those that were located included a limited range of nutrition risk indicators such as weight, height and biochemistry^(24)^. Ultimately, a well-trained system could reliably identify at-risk patients by mining electronic medical records and hospital databases in order to triage patients, on a real-time basis, for dietetic screening, assessment and management. Such a system would represent an opportunity to optimise the use of limited dietetics resources, which has already been identified as a significant barrier to diagnosing and treating malnutrition in the acute setting^(1)^.

The findings of this review of reviews are limited by the lack of eligible high-quality reviews available for inclusion and need to be considered in this context. It is possible that other risk factors exist that to date have not yet been captured in a systematic review and hence are not included in our list of indicators. In addition, some of the reviews^(17,18)^ were limited by a lack of relevant original articles for inclusion. However, the large number of reviews excluded after quality assessment highlights the need for rigorous study design in research in order that findings can be interpreted with confidence. It was also difficult to make comparisons across the included reviews due to their heterogeneity in terms of indicators investigated, outcome measures reported and data analysis techniques used. This also prohibited the ranking of indicators according to the magnitude of impact on nutrition risk, as well as the potential interplay between different indicators. Furthermore, the reviews included in this narrative review were from seven different countries. While this may be a limitation given the multifactorial nature of malnutrition, these countries represent a broad cross-section of developed countries, thus this may increase the generalisability of findings to the developed world. Finally, each review investigated potential indicators in a different inpatient population (e.g. stroke patients, cancer patients etc.), meaning that the factors listed in our comprehensive list may not predict nutritional risk in all patient populations. Nonetheless, to our knowledge, this is the first attempt to synthesise the abundance of literature on the subject of malnutrition with the aim to generate a thorough list of nutritional risk factors identified from high-quality literature, following a rigorous systematic methodology.

## Conclusion

The findings of this review represent the first comprehensive list of risk factors for malnutrition in the acute care setting that is based only on high-quality evidence. The availability of this comprehensive list has the potential to optimise the use of dietetics resources and patient outcomes through the development of novel, holistic, automated, systems-based screening pathways that may be better able to identify and triage patients according to nutritional risk than current tools. In order to develop such novel approaches, more research is needed to identify the magnitude of risk that can be attributed to each of the identified indicators and the interplay between them. While the literature is an important component, clinical expertise is also important and future research will include a consideration of both aspects in the development of systems-level approaches.

## Supplementary material

For supplementary material accompanying this paper visit https://doi.org/10.1017/jns.2021.86. click here to view supplementary material

## References

[1] Barker LA, Gout BS & Crowe TC (2011) Hospital malnutrition: prevalence, identification and impact on patients and the healthcare system. Int J Environ Res Public Health 8, 514–527. 21556200 10.3390/ijerph8020514 PMC3084475

[2] Allard JP, Keller H, Jeejeebhoy KN, (2016) Decline in nutritional status is associated with prolonged length of stay in hospitalized patients admitted for 7 days or more: a prospective cohort study. Clin Nutr 35, 144–152. 25660316 10.1016/j.clnu.2015.01.009

[3] Saunders J (2010) Malnutrition: causes and consequences. Clin Med (Lond) 10, 624–627. 21413492 10.7861/clinmedicine.10-6-624 PMC4951875

[4] Food and Agriculture Organisation of the United Nations (2013) State of Food and Agriculture. Rome, Italy: FAO.

[5] Miller J, Wells L, Nwulu U, (2018) Validated screening tools for the assessment of cachexia, sarcopenia, and malnutrition: a systematic review. Am J Clin Nutr 108, 1196–1208. 30541096 10.1093/ajcn/nqy244

[6] Baker JP, Detsky AS, Wesson DE, (1992) Nutritional assessment: a comparison of clinical judgement and objective measurements. N Engl J Med 306, 969–972. 10.1056/NEJM198204223061606 6801515

[7] Elia M (editor) (2003) *The ‘MUST’ Report: Nutritional Screening of Adults: A Multidisciplinary Responsibility. Development and Use of the ‘Malnutrition Universal Screening Tool’ (‘MUST’) for Adults*. Redditch, UK: A Report by the Malnutrition Advisory Group of the British Association for Patenteral and Enteral Nutrition.

[8] Cant RP (2011) Investing in patients’ nutrition: nutrition risk screening in hospital. Aust J Adv Nurs 28, 81–87.

[9] Porter J, Raja R, Cant T, (2009) Exploring issues influencing the use of the Malnutrition Universal Screening Tool by nurses in two Australian hospitals. J Hum Nutr Diet 22, 203–209. 19175489 10.1111/j.1365-277X.2008.00932.x

[10] Raja R, Gibson S, Turner A, (2008) Nurses’ views and practices regarding use of validated nutrition screening tools. Aust J Adv Nurs 26, 26–33.

[11] Porter J & Jamieson R (2013) Triaging in dietetics: do we prioritise the *right patients?* Nutr Diet 70, 21–26.

[12] Phillips W & Zechariah S (2017) Minimizing false-positive nutrition referrals generated from the Malnutrition Screening Tool. J Acad Nutr Diet 117, 665–669. 27421778 10.1016/j.jand.2016.05.014

[13] Raynaud-Simon A, Reval-Delhom C & Hebuterne X (2011) Clinical practice guidelines from the French Health High Authority: nutritional support strategy in protein-energy malnutrition in the elderly. Clin Nutr 30, 312–319. 21251732 10.1016/j.clnu.2010.12.003

[14] Covidence. Covidence Systematic Review Software. Melbourne, Australia: Veritas Health Innovation; available at http://www.covidence.org.

[15] Academy of Nutrition and Dietetics (2016) Evidence Analysis Manual: Steps in the Academy Evidence Analysis Process. https://www.andeal.org/vault/2440/web/files/2016_April_EA_Manual.pdf.

[16] Chen N, Li Y, Fang J, (2019) Risk factors for malnutrition in stroke patients: a meta-analysis. Clin Nutr 38, 127–135. 29310894 10.1016/j.clnu.2017.12.014

[17] Fruchtenicht AVG, Poziomyck AK, Kabke GB, (2015) Nutritional risk assessment in critically ill cancer patients. Rev Bras Ter Intensiva 27, 274–283. 26270855 10.5935/0103-507X.20150032 PMC4592123

[18] Lew CCH, Ong F & Miller M (2016) Validity of the adductor pollicis muscle as a component of nutritional screening in the hospital setting: a systematic review. Clin Nutr ESPEN 16, 1–7. 28531449 10.1016/j.clnesp.2016.08.005

[19] Miller IM, Ellervik C, Yazdanyar S, (2013) Meta-analysis of psoriasis, cardiovascular disease, and associated risk factors. J Am Acad Dermatol 69, 1014–1024. 24238156 10.1016/j.jaad.2013.06.053

[20] Perry L & Love CP (2001) Screening for dysphagia and aspiration in acute stroke: a systematic review. Dysphagia 16, 7–18. 11213249 10.1007/pl00021290

[21] Tenner S, Baillie J, Dewitt J, (2013) American college of gastroenterology guideline: management of acute pancreatitis. Am J Gastroenterol 108, 1400–1415. 23896955 10.1038/ajg.2013.218

[22] National Health and Medical Research Council (2009) NHMRC Levels Of Evidence and Grades for Recommendations for Guideline Developers. https://www.nhmrc.gov.au/_files_nhmrc/file/guidelines/developers/nhmrc_levels_grades_evidence_120423.pdf (accessed May 2019).

[23] Ferguson M, Capra S, Bauer J, (1999) Development of a valid and reliable malnutrition screening tool for adult acute hospital patients. Nutrition 15, 458–464. 10378201 10.1016/s0899-9007(99)00084-2

[24] Trtovac D & Lee J (2018) The use of technology in identifying hospital malnutrition: scoping review. JMIR Med Inform 6, e4. 29351894 10.2196/medinform.7601 PMC5797288

