# Supplementary material for: Indicators of nutritional risk in hospital inpatients: a narrative review
Source: J Nutr Sci. 2021 Dec 10;10:e104. doi: 10.1017/jns.2021.86 (PMC8727709; doi:10.1017/jns.2021.86)
Supplement: Supplementary file 1 [file S2048679021000860sup001.docx]

**Supplemental Table 1: Medline search strategy for a literature review to identify reviews reporting nutritional risk indicators in adult acute care inpatients**

| **#** | **Searches** |
| --- | --- |
| 1 | hospitalization/ or inpatients/ |
| 2 | hospital units/ or intensive care units/ or burn units/ or coronary care units/ |
| 3 | acute disease/ or critical illness/ |
| 4 | (hospital* or inpatient*).tw,kw. |
| 5 | (patient* adj4 (admitted or admission*)).tw,kw. |
| 6 | ((critical* or acute*) adj4 (ill* or episode* or disease* or care)).tw,kw. |
| 7 | (ICU or ICUs or ((intensive care or burns or coronary care) adj2 (unit* or ward*))).tw,kw. |
| 8 | or/1-7 |
| 9 | Risk factors/ or Risk/ or Risk assessment/ |
| 10 | (Risk* or determinant* or vulnerab* or predispos* or susceptib* or danger*).tw,kw. |
| 11 | 9 or 10 |
| 12 | nutrition disorders/ or nutritional requirements/ or Nutritional Physiological Phenomena/ or Elder nutritional Physiological Phenomena/ |
| 13 | (nutrition* adj4 (disorder* or disease* or risk* or vulnerab* or compromised or abnormal* or impair* or complication* or deplet* or insufficien* or deficien* or complication* or derange* or stress or require*)).tw,kw. |
| 14 | overnutrition/ or exp obesity/ |
| 15 | (overnutrition or overnourish* or hypernutrition or overweight or obes*).tw,kw. |
| 16 | malnutrition/ or protein-energy malnutrition/ or severe acute malnutrition/ or wasting syndrome/ or hiv wasting syndrome/ or refeeding syndrome/ or severe acute malnutrition/ or kwashiorkor/ or starvation/ |
| 17 | cachexia/ or sarcopenia/ or emaciation/ |
| 18 | (nourish* or undernutrition or undernourish* or malnutrition* or malnourish* or wasting or emaciat* or cachexia or cachectic or sarcopeni* or kwashiorkor or starv*).tw,kw. |
| 19 | hypervitaminosis a/ or avitaminosis/ |
| 20 | (hypervitaminosis or avitaminosis).tw,kw. |
| 21 | (micronutrients/ or ubiquinone/ or trace elements/ or iodine/ or iron/ or selenium/ or zinc/ or vitamins/ or 25-hydroxyvitamin d 2/ or ascorbic acid/ or vitamin a/ or vitamin e/ or vitamin b complex/ or biotin/ or folic acid/ or niacin/ or pantothenic acid/ or pyridoxine/ or riboflavin/ or thiamine/ or vitamin b 12/ or vitamin b 6/ or calcium/) and deficiency.fs. |
| 22 | deficiency diseases/ |
| 23 | ascorbic acid deficiency/ or scurvy/ or vitamin a deficiency/ or vitamin b deficiency/ or choline deficiency/ or folic acid deficiency/ or hyperhomocysteinemia/ or pellagra/ or riboflavin deficiency/ or thiamine deficiency/ or beriberi/ or wernicke encephalopathy/ or vitamin b 6 deficiency/ or vitamin b 12 deficiency/ or anemia, pernicious/ or subacute combined degeneration/ or vitamin d deficiency/ or rickets/ or osteomalacia/ or "chronic kidney disease-mineral and bone disorder"/ or rickets, hypophosphatemic/ or vitamin e deficiency/ or vitamin k deficiency/ or magnesium deficiency/ or potassium deficiency/ or protein deficiency/ |
| 24 | ((vitamin* or multivitamin* or micronutrient* or macronutrient* or nutrient* or trace element* or mineral* or magnesium or zinc or selenium or potassium or ascorbic acid or choline or folic acid* or folate or riboflavin or thiamine or protein or calcium or iron or iodine) adj4 (deficien* or deplet* or level* or inadequa* or insufficien* or lacking)).tw,kw. |
| 25 | (scurvy or hyperhomocysteinemia or pellagra or rickets or refeeding syndrome* or beriberi or osteomalacia or wernicke*).tw,kw. |
| 26 | ((Pernicious or Biermer* or Addison*) adj2 (anemi* or anaemi*)).tw,kw. |
| 27 | (iron adj3 (anemi* or anaemi*)).tw,kw. |
| 28 | ((B12 or B-12) adj3 (anemi* or anaemi*)).tw,kw. |
| 29 | (micronutrients/ or ubiquinone/ or trace elements/ or iodine/ or iron/ or selenium/ or zinc/ or vitamins/ or 25-hydroxyvitamin d 2/ or ascorbic acid/ or vitamin a/ or vitamin e/ or vitamin b complex/ or biotin/ or folic acid/ or niacin/ or pantothenic acid/ or pyridoxine/ or riboflavin/ or thiamine/ or vitamin b 12/ or vitamin b 6/ or calcium/) and (adverse effects or toxicity or poisoning).fs. |
| 30 | ((vitamin* or multivitamin* or micronutrient* or macronutrient* or nutrient* or trace element* or mineral* or magnesium or zinc or selenium or potassium or ascorbic acid or choline or folic acid* or folate or riboflavin or thiamine or protein or calcium or iron or iodine) adj4 (toxicit* or poisoning)).tw,kw. |
| 31 | Hypocalcemia/ |
| 32 | Hypocalcemia/ or (hypocalcemi* or hypocalcaemi*).tw,kw. |
| 33 | Dehydration/ |
| 34 | (dehydration or water stress).tw,kw. |
| 35 | or/12-34 |
| 36 | 8 and 11 and 35 |
| 37 | (meta analysis or systematic review or review).pt. |
| 38 | meta-analysis/ |
| 39 | meta-analysis as topic/ |
| 40 | "Review Literature as Topic"/ |
| 41 | Technology Assessment, Biomedical/ |
| 42 | ((systematic* adj3 (review* or overview*)) or (methodologic* adj3 (review* or overview*))).tw,kw. |
| 43 | ((quantitative adj3 (review* or overview* or synthes*)) or (research adj3 (integrati* or overview*))).tw,kw. |
| 44 | ((integrative adj3 (review* or overview*)) or (collaborative adj3 (review* or overview*)) or (pool* adj3 analy*)).tw,kw. |
| 45 | (data synthes* or data extraction* or data abstraction*).tw,kw. |
| 46 | (handsearch* or hand search*).tw,kw. |
| 47 | (mantel haenszel or peto or der simonian or dersimonian or fixed effect* or latin square*).tw,kw. |
| 48 | (met analy* or metanaly* or technology assessment* or HTA or HTAs or technology overview* or technology appraisal*).tw,kw. |
| 49 | (meta regression* or metaregression*).tw,kw. |
| 50 | (meta-analy* or metaanaly* or systematic review* or biomedical technology assessment* or bio-medical technology assessment*).mp,hw. |
| 51 | (medline or cochrane or pubmed or medlars or embase or cinahl).tw,hw. |
| 52 | (cochrane or (health adj2 technology assessment) or evidence report).jw. |
| 53 | (comparative adj3 (efficacy or effectiveness)).tw,kw. |
| 54 | (outcomes research or relative effectiveness).tw,kw. |
| 55 | ((indirect or indirect treatment or mixed-treatment) adj comparison*).tw,kw. |
| 56 | or/37-55 |
| 57 | 36 and 56 |
| 58 | exp animals/ not humans/ |
| 59 | (exp child/ or exp infant/ or adolescent/) not exp adult/ |
| 60 | (child* or preschool* or toddler* or p?ediatric* or adolescen* or youth or teen*).ti. |
| 61 | (mice or mouse or murine or rat or rats or pig or swine or rabbit* or equine or horse*).ti. |
| 62 | or/58-61 |
| 63 | 57 not 62 |
| 64 | limit 63 to english language |

**Notes**

- Ovid MEDLINE(R) Epub Ahead of Print, In-Process & Other Non-Indexed Citations, Ovid MEDLINE(R) Daily, Ovid MEDLINE and Versions(R)
- / = search on Medical Subject Headings (fixed terms)
- Tw,kw = search on title, abstract, and author keyword fields
- Adjx allows terms to occur within x spaces of each other in either direction (i.e. *patients admitted* or *admitted patients*)
- Fs = free floating subheading (i.e. a MeSH subheading allowed to attach itself to any MeSH term in the search)
- Pt = search on publication type (fixed terms)
- ? substitutes for 0-1 characters, e.g. *P?ediatric* finds *pediatric* or *paediatric*
